# Delaying Early Morning Training Time in Swimmers: Effects on Sleep, Recovery and Performance—A Randomized Controlled Trial

**DOI:** 10.1186/s40798-026-01086-x

**Published:** 2026-07-28

**Authors:** Maxime M. E. Brandts, Sabrina Forster, Anne Hecksteden, Tim Meyer

**Affiliations:** 1 Institute of Sports and Preventive Medicine, Saarland University, Saarbrücken, Germany; 2 Institute of Sport Science, University of Innsbruck, Innsbruck, Austria; 3 Institute of Physiology, Medical University of Innsbruck, Innsbruck, Austria

**Keywords:** National level swimmers, Sleep extension, Sleep duration, Actigraphy, Perceived recovery, Training camp, Mediation analysis

## Abstract

**Background:**

Swimmers often fail to achieve recommended sleep durations. Early morning training, common in swimming, is associated with shorter sleep. Sleep extension through later wake-up times appears to be an appropriate measure to counteract this. Therefore, this study investigated whether delaying early morning training has an effect on sleep, recovery and performance, and whether changes in sleep mediate eventual effects on recovery and performance.

**Methods:**

Using cross-over design, 27 national-level swimmers (13 females, 14 males; age: 15.6 ± 1.0 years) completed two 8-day training camps in January and October 2023 in Saarbrücken, Germany. Morning sessions started at 07:00 AM (early) or 09:00 AM (late), with conditions randomized between camps. Sleep and recovery were assessed using actigraphy, sleep diaries, and the Short Recovery and Stress Scale. Performance was evaluated using 100 m main stroke and 800 m freestyle time-trials pre- and post-camp. Linear mixed-effects models and repeated measures analysis of variance were employed to assess the effect of training time on sleep, recovery and performance. Regression-based mediation analysis tested whether changes in sleep and recovery mediated the effects of training timepoint on recovery and performance.

**Results:**

Sleep duration per night was significantly longer in the late compared to the early condition (1.0 ± 0.5 h; t = 14.0, *p* < .001). However, there was no significant effect on measures of recovery (*p* > .080), and 100 m and 800 m performance (*p* > .204). Mediation analysis showed no indirect effects of sleep duration on mental capacity or of mental capacity on performance (*p* > .05).

**Conclusion:**

Delaying early morning training from 07:00 AM to 09:00 AM increased sleep duration by one hour on average, but this did not significantly affect recovery or performance in elite swimmers. Nevertheless, the study provides evidence that early morning training is associated with reduced sleep duration and highlights the persistent sleep challenges faced by adolescent swimmers.

*Trial registration DRKS,* DRKS00039267*. Registered 17 February 2026—Retrospectively registered, *https://www.drks.de/DRKS00039267*.*

## Background

Sleep plays a crucial role in multiple restorative and developmental processes across the nervous, metabolic, musculoskeletal, and endocrine systems [1]. Accordingly, sleep loss can have wide-ranging consequences, impairing attention, working memory, decision-making, learning capacity, and mood regulation [2], while also increasing susceptibility to infections, inflammatory diseases [3], and reduced muscle protein synthesis [4].

A common form of sleep loss is sleep restriction, also referred to as partial sleep deprivation, which occurs when sleep duration is restricted below an individual’s sleep need, without complete absence of sleep [5]. Sleep restriction has been shown to negatively influence sporting performance, including anaerobic power, speed/power endurance, high-intensity interval exercise, maximal strength, strength-endurance, and skill execution [6]. Notably, when sleep is restricted at the end of the night (i.e., early wake-up times), referred to as "late sleep restriction", the performance impairments appear to be particularly pronounced [6–8]. Together, these findings suggest that sleep loss can be detrimental to performance, particularly when sleep is restricted in the morning, as with early morning training.

While the effects of sleep restriction on performance are well documented, evidence regarding its impact on recovery is limited. Rae et al. [9], observed that recovery from high-intensity interval training was impaired after one night of sleep restriction. These findings, together with the cognitive and physiological consequences, suggest a potential role of sleep for recovery. Accordingly, coaches and athletes recognize sleep as a key component of recovery [10–12].

Despite this recognition, previous research indicates that athletes often fail to obtain recommended sleep durations of 7–9 h for adults and 8–10 h for adolescents [13–15]. Sargent et al. [16] further reported that athletes not only sleep less than these recommendations but also less than they believe they need, with an average perceived sleep deficit of 96 min per night.

This shortfall appears to be even more pronounced in swimmers, with reported sleep durations ranging from 6.2 to 7.7 h [16–19], and an average perceived sleep deficit of 118 min [16]. Previous studies suggest that the prevalence of early morning training in swimming may contribute to this, with evidence showing that sleep on nights preceding sessions starting before 07:00 AM is reduced by 1–2 h compared to nights before later sessions or rest days [17, 18, 20]. Considering the impairments observed with (late) sleep restriction [6–9], early morning training may similarly compromise performance and recovery.

To mitigate these effects, several strategies have been proposed, such as napping, light therapy, cold water immersion and sleep extension [21]. Among these, sleep extension appears particularly promising, with a recent systematic review demonstrating consistent improvements in performance outcomes when sleep was prolonged by 46–113 min beyond a habitual duration of ~7 h, resulting in total sleep duration >8 h [21].

While sleep extension appears to improve performance, its effects on post-exercise recovery remain to be investigated. Additionally, although early morning training has been linked to reduced sleep duration, existing studies are observational in nature, limiting conclusions about causality [17, 18, 20]. Consequently, it remains unknown whether delaying early morning training can extend sleep duration and, enhance recovery and performance. A training camp would provide a controlled setting to investigate this, and is particularly relevant as previous studies suggest that periods of high training load can affect sleep duration and sleep quality [22–25].

Therefore, the aim of this study was to investigate whether delaying early morning training during a training camp influences sleep, perceived recovery, and swimming performance. Beyond examining these outcomes in isolation, this study explored whether delaying morning training affects recovery through changes in sleep, and whether it affects swimming performance through changes in recovery.

## Methods

### Participants

Thirty-four tier 3 level swimmers (17 females, 17 males, age: 16 ± 1 years) participated in this study [26]. Recruitment was conducted through an invitation by the German national swimming federation to a training camp. An a priori sample size calculation for the Lamberts and Lambert Submaximal Cycling Test (LSCT; d = 0.2, α = 0.05, power = 0.80) indicated a required sample of 155. Since this was not practically feasible, a target of 30 was set, at which medium effects could be detected (d = 0.46). As the LSCT was not a primary outcome here, a simulation-based power analysis for the reported outcomes is provided (Sect. "Data Analysis"). Prior to the study, participants received detailed information about its objectives and procedures, and provided written informed consent. For participants under 18 years of age, written informed consent was also obtained from their parents or legal guardians.

Prior to the start of the study, participants were screened for sleep disorders using the Sleep Questionnaire B/Revised (SF-B/R; Görtelmeyer, 2011), and chronotype was assessed using the Morningness–Eveningness Questionnaire (MEQ; Horne & Östberg, 1976 [27]). Swimmers were excluded from participation in the case of an injury or medical condition that precludes physical exertion, or in the case of a clinically diagnosed sleep disorder. The study was conducted in accordance with the Declaration of Helsinki, with the exception that it was retrospectively registered at the clinical trial registry. The study was approved by the local Human Research Ethics Committee (Ärztekammer des Saarlandes, Saarbrücken, Germany, registration no.: 114/22).

### Study Design

Two 8-day training camps were conducted in January 2023 and October 2023 at the Olympic training center in Saarbrücken, Germany. All training took place on-site, and participants resided in shared on-site accommodation, away from their habitual sleeping environment. Each camp included a total of 13 swimming sessions, consisting of 6 morning and 7 afternoon sessions. The first and final sessions took place in the afternoon of day 1 and day 7, respectively, with no training scheduled on day 8. Morning training sessions began either at 07:00 AM (early condition) or 09:00 AM (late condition). Participants were individually randomized to one of the two conditions, with allocation reversed in the second camp to allow for within-subject comparisons. Randomization was performed using a random number generator in Microsoft Excel without stratification. Both training camps were designed and organized by the same head coach, who kept the structure and content of the training sessions consistent across both conditions and camps. Swimming performance, recovery status and motivation were assessed on day 1 (pre-test) and day 8 (post-test). Daily measures of sleep, perceived recovery, and training load were collected during the two weeks preceding each camp as well as throughout the entire training camp.

### Measurements

#### Performance

Swimming performance was assessed in a 50 m indoor pool through a 100 m time-trial in each participant’s main stroke and an 800 m freestyle time-trial. Participants were divided into series of 4–5 swimmers based on main stroke and personal best times. Time-trial performance was measured manually by experienced coaches using handheld stopwatches. Before the 100 m time-trial, participants completed a standardized warm-up consisting of 2 × 500 m and 1 × 200 m freestyle at 60%, 80% and 90% intensity, respectively, based on the Lamberts & Lambert Submaximal Cycling Test [28]. Time between the warm-up, the 100 m time-trial and the 800 m time-trial were 10–20 and 60–85 min, respectively, to ensure sufficient recovery. The start time of performance testing varied by condition: the early group began between 07:00 AM and 07:45 AM, while the late group started between 09:00 AM and 10:00 AM. Participants were instructed to replicate their nutritional intake during the 24 h preceding the post-test to match that of the 24 h prior to the pre-test, as recorded in a nutrition protocol. Breakfast prior to training and performance testing was provided on-site for all participants.

#### Sleep

Nocturnal sleep duration, sleep onset latency, sleep efficiency, and perceived sleep quality were assessed using actigraphy (BT-WGT3X ActiGraph, ActiGraph LLC, Pensacola, FL, USA) in combination with a sleep diary. Participants were instructed to wear the actigraph on their non-dominant wrist throughout the study period, except during water-based activities.

Actigraphy data were processed using the GGIR package (version 3.1–5) [29], which has been previously validated against polysomnography [30]. The sleep diary was used to define the sleep period time window [31]. In cases where sleep diary data were unavailable, sleep timing was estimated using the HDCZA algorithm [32], implemented within GGIR.

Single nights were excluded from analysis if less than eight hours of valid wear data were recorded between noon and noon the following day, to account for potential non-wear periods before and after bedtime despite instructions to the contrary. Sleep periods containing more than 20% non-wear time were excluded, based on exploratory analyses showing increased variability in sleep measures beyond this threshold. Additionally, any night where the recorded wake-up time occurred after the scheduled morning training start was omitted.

At the start of the study, participants were instructed to abstain from napping throughout the training camp. However, on day 3, the coaching staff raised concerns about an expected high drop-out rate due to accumulated fatigue. Considering this and the common use of napping in training camps, daytime naps were permitted from day 3 onward in both camps and recorded using a nap diary.

#### Recovery and Training

Measures of recovery were obtained during the standardized warm-up prior to performance testing and through daily self-report. After each stage of the warm-up, athletes reported their rate of perceived exertion (RPE) and heart rate was assessed by palpation of the carotid artery for 10 s. Perceived recovery and stress were assessed each morning using the Short Recovery and Stress Scale (SRSS), an 8-item questionnaire [33]. Training load was assessed in the evenings, athletes reported training characteristics, including session duration and session rate of perceived exertion (sRPE). Session load was calculated as the product of sRPE and session duration, and a 7-day rolling average was computed to monitor training load over time. Data from days on which participants reported illness during the camp were excluded from analyses, as illness may affect both perceived recovery and stress. SRSS and RPE were collected via a custom, GDPR conforming smartphone application as previously published [34].

### Data Analysis

Descriptive statistics were used to summarize participant characteristics, including sex, age, and chronotype distribution. Baseline sleep and chronic training load were compared between conditions (early, late) using repeated measures analysis of variance (ANOVA), to assess potential differences. Daily session load during the training camps was analyzed with a linear mixed-effects model including participant as a random intercept to assess whether training was comparable. To examine potential period effects, paired-sample t-tests were performed to compare the first and second camp in baseline average sleep, baseline chronic training load, average camp session load and pre-camp performance.

The effect of condition (early, late) on sleep, napping, recovery, performance, and motivation was first assessed in isolation. Linear mixed-effects models were used for sleep, nap duration, 24-h sleep duration and perceived recovery, and a Poisson mixed-effects model for nap count. Changes in heart rate and RPE during the standardized warm-up were analyzed using a two-way repeated measures ANOVA with intensity (60%, 80%, 90%), and condition (early, late) as within-participant factors. Performance and motivation were analyzed with two-way repeated measures ANOVA with timepoint (pre, post) and condition (early, late) as within-participant factors.

To assess whether the effect of delaying morning training on recovery and swimming performance operated through changes in sleep and subsequent recovery, a regression-based mediation analysis was conducted. Mediation analysis examines whether the effect of an independent variable (X) on a dependent variable (Y) occurs directly (X → Y) or indirectly through one or more intermediate variables (X  →  M  →  Y), referred to as mediators (M).

**Fig.1:**
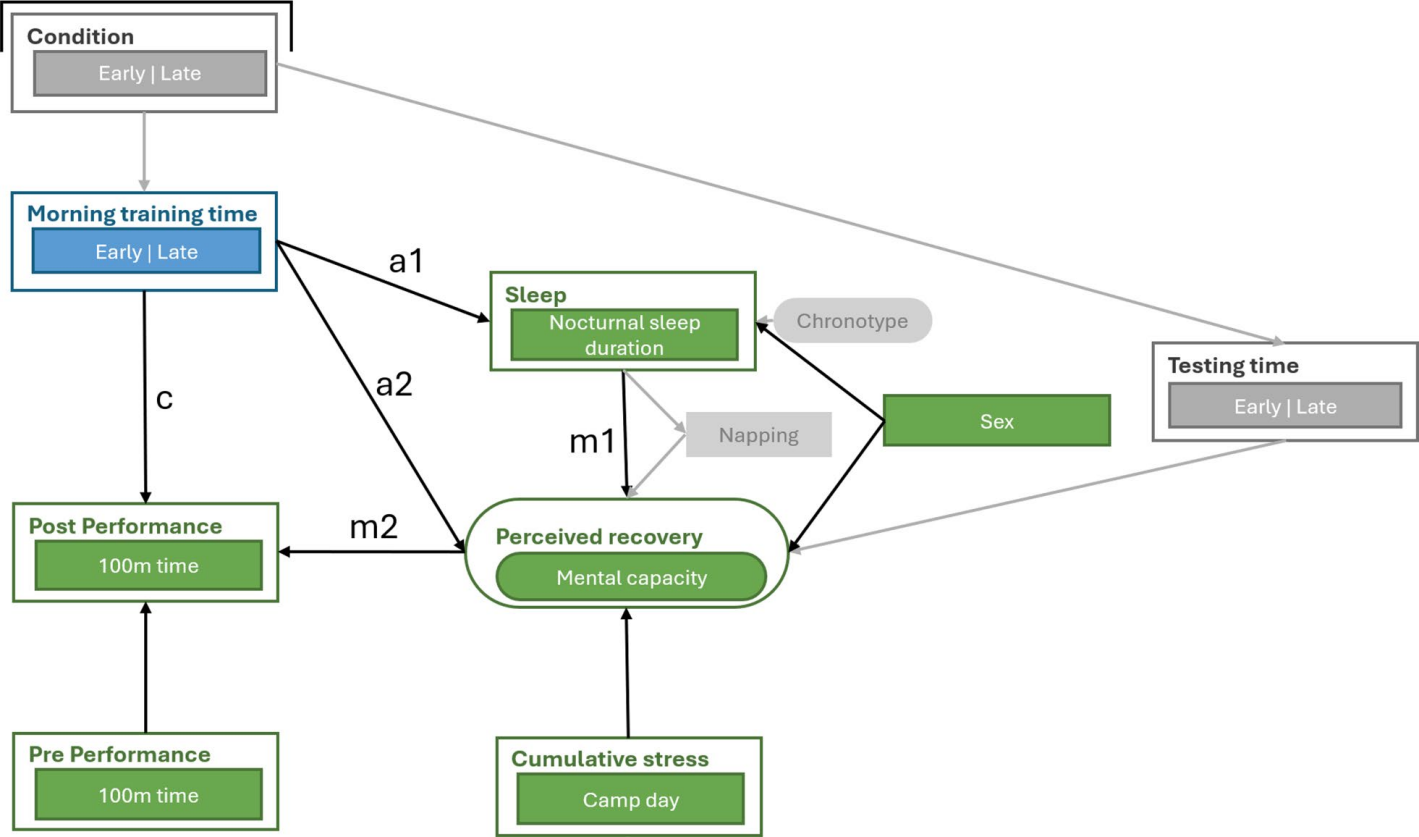
Directed acyclic graph representing the hypothesized mediation pathways linking morning training time (blue) to sleep, recovery and performance (green), directly or indirectly. Grey-shaded variables were considered conceptually but not included in the statistical mediation analysis. Arrows indicate the hypothesized direction of causal effects

The hypothesized pathway is illustrated in a directed acyclic graph (DAG; Fig. 1), where arrows represent assumed causal directions. In this model, sleep, recovery, and performance are operationalized as sleep duration, mental capacity, and 100 m time, respectively.

Each pathway in the DAG was evaluated using linear models:

1. Sleep duration = *a*_*1*_ x condition x sex,
2. Mental capacity = *m*_*1*_ x sleep duration x sex + *a*_*2*_ x condition x camp day,
3. Post 100 m time = *m*_*2*_ x mental capacity + *c* x condition + pre 100 m time.

Random intercepts and slopes for participants were included in models 1 and 2. For model 3, only mental capacity measures obtained on the same day as the post-performance assessment were considered.

Exclusion of participants due to missing data was applied at two levels: general and outcome specific. First, participants who did not complete both training camps were excluded from all analyses. Second, within each outcome variable, participants with fewer than two observations were excluded from the corresponding analysis. As a result, missing data on one outcome did not preclude inclusion in analyses of other outcomes maximizing the use of available data.

A simulation-based power analysis was conducted to evaluate the ability to detect differences in sleep duration between the early and late condition, given the observed sample structure. The alpha level was set at 0.05, and 100 simulations were performed. Fixed-effect estimates and variance components were based on Ashby et al. (2024) [17]. This yielded an estimated power of 100% (95% CI 96–100%).

All analyses were conducted in R. (version 4.3.3). The corresponding code and data are available at https://github.com/MBrandts98/SleepTrain_analysis Results are reported as mean ± standard deviation or as unstandardized coefficients (b) with standard errors (SE), accompanied by relevant statistical estimates from the mixed-effects models or ANOVA, as appropriate.

## Results

### Participant Characteristics

**Fig.2:**
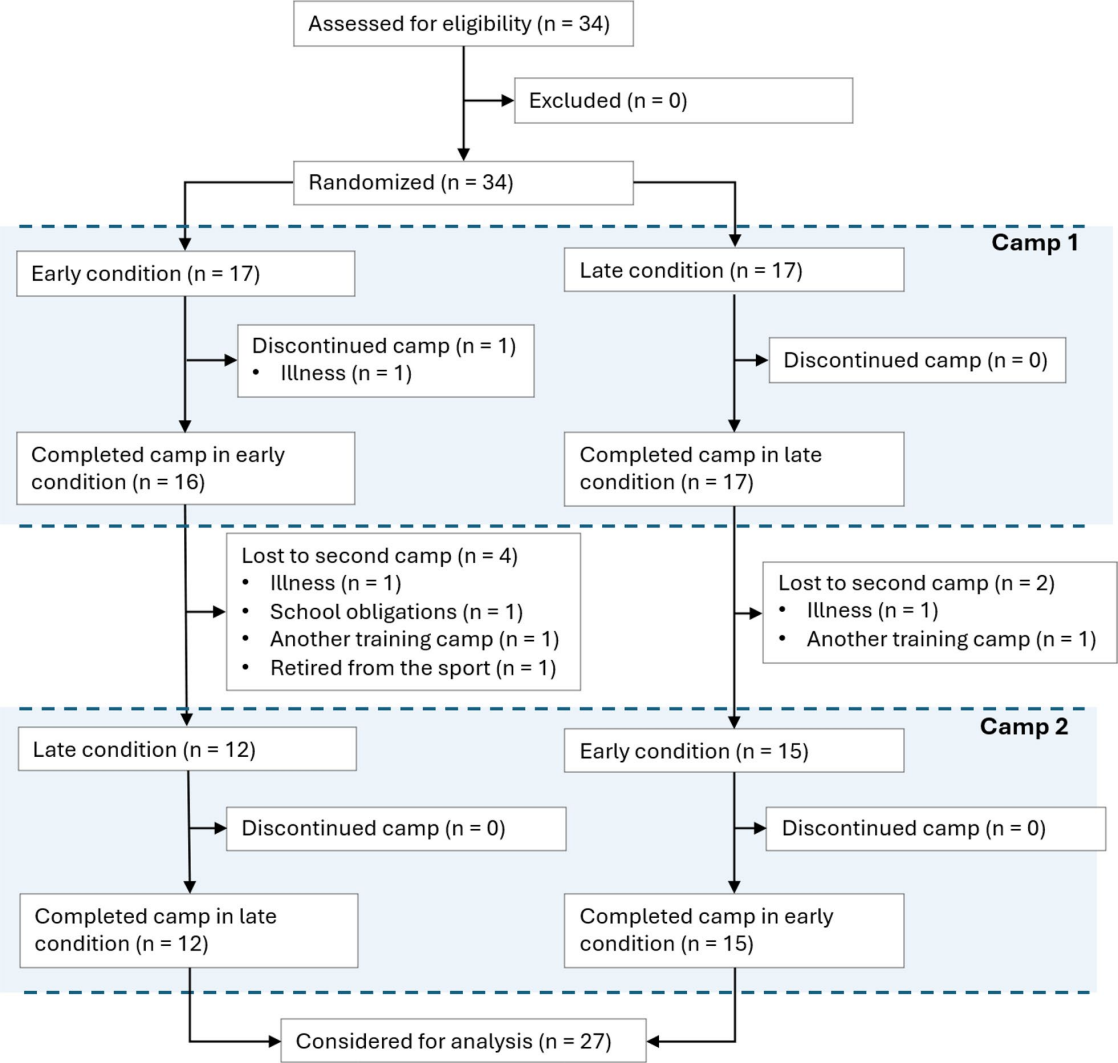
Participant flow throughout the study

**Fig.3:**
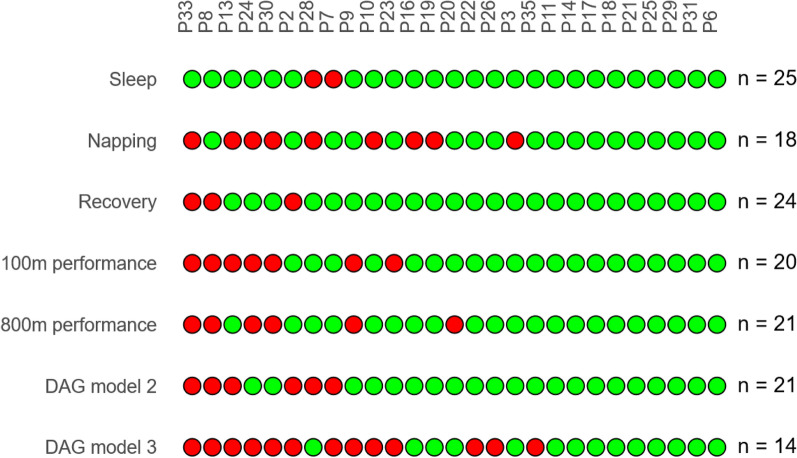
Participant inclusion across analyses. Analyses are listed on the left y-axis, participant codes on the x-axis (top), and the number of participants included per analysis on right. Green dots indicate inclusion, red dots exclusion. *DAG* directed acyclic graph

Participant flow across the study is depicted in Fig. 2. Of the 34 athletes who took part in the first training camp, 27 completed both camps and were considered for the analyses (13 females, 14 males; age: 15.6 ± 1.0 years). No participants were excluded based on the SF-B/R. Chronotype distribution, based on self-report, was predominantly neutral (*n* = 21), with three participants classified as rather morning types and three as rather evening types. The specific analyses in which participants were included are illustrated in Fig. 3, resulting in samples ranging from 14 to 25 swimmers.

### Baseline Characteristics and Training Load

No statistically significant differences were found between the early and late condition in baseline sleep parameters (bedtime, waketime, and sleep duration) or chronic training load (*p* > 0.20). Additionally, session load during the training camps did not differ significantly between conditions (*p* = 0.47). Detailed values and test statistics comparing the early and late conditions are reported in Table 1. No significant condition x sex interaction effects or main effects of sex were observed (p > 0.15).

**Table 1:** Summary of baseline characteristics, training load, sleep and recovery outcomes across the early and late training conditions

	Early	Late	Late vs. early ^a^		
	Mean ± SD	Mean ± SD	Mean ± SD	t-value	*p*-value
Baseline					
Average bedtime (hh:mm)	23:00 ± 01:00	23:13 ± 00:49	00:13 ± 00:54	1.33	.199
Average waketime (hh:mm)	06:50 ± 01:12	06:59 ± 01:04	00:08 ± 01:11	0.68	.503
Average sleep duration (h)	6.6 ± 0.8	6.6 ± 0.7	− 0.0 ± 1.2	− 0.09	.923
7-day average session load ^b^	435 ± 255	481 ± 272	46 ± 359	0.67	.506
Training					
Average daily session load ^b^	1005 ± 258	964 ± 296	− 40 ± 305	− 0.73	.465
Sleep					
Average bedtime (hh:mm)	22:54 ± 00:28	23:18 ± 00:32	00:24 ± 00:22	6.25	< .001
Average waketime (hh:mm)	05:36 ± 00:09	07:18 ± 00:25	01:43 ± 00:28	40.59	< .001
Sleep onset latency (min)	25 ± 8	26 ± 13	1 ± 14	0.26	.794
Number of naps	2.3 ± 1.7	1.3 ± 1.1	− 1 ± 1.7	− 2.22	.023
24-h sleep duration (h)	6.5 ± 0.6	7.3 ± 0.5	0.8 ± 0.6	4.28	< .001
Average nap duration (min)	46 ± 37	42 ± 36	− 4 ± 57	− 0.65	.521
Recovery ^c^					
∆ HR @ 60% (bpm)	− 1 ± 16	− 8 ± 13	− 7 ± 21	− 1.87	.080
∆ HR @ 80% (bpm)	− 1 ± 15	− 7 ± 17	− 6 ± 26	− 1.32	.202
∆ HR @ 90% (bpm)	− 1 ± 16	− 4 ± 19	− 4 ± 24	− 0.64	.532
∆ RPE @ 60%	− 0.6 ± 5.1	0.0 ± 1.2	0.7 ± 5.2	0.54	.594
∆ RPE @ 80%	− 0.3 ± 4.9	0.7 ± 1.5	1.0 ± 4.7	0.86	.401
∆ RPE @ 90%	0.1 ± 3.1	1.1 ± 3.3	1.0 ± 3.7	0.99	.331
General recovery	2.6 ± 0.8	2.8 ± 0.6	0.2 ± 0.6	1.37	.184
Physical capacity	3.0 ± 0.8	3.1 ± 0.8	0.1 ± 0.7	0.62	.538
Mental capacity	3.2 ± 0.8	3.5 ± 0.9	0.3 ± 0.9	1.81	.084
Emotional balance	3.6 ± 1.0	3.8 ± 1.0	0.2 ± 0.9	1.16	.261
General stress	2.9 ± 1.3	3.0 ± 1.1	0.1 ± 1.6	0.44	.664
Muscular stress	3.0 ± 1.2	3.0 ± 1.0	0.1 ± 1.3	0.27	.793
Lack of activation	2.4 ± 1.4	2.0 ± 1.1	− 0.1 ± 1.6	− 1.05	.305
Emotional disbalance	1.8 ± 1.3	1.7 ± 1.1	− 0.4 ± 1.2	− 0.19	.855

^a^The final column presents the mean difference (late–early), associated *t*-values, and *p*-values for paired comparisons

^b^Session load was calculated as session RPE multiplied by session duration

^c^Measures of perceived recovery are provided on a scale from 0 to 6

^d^*SD* standard deviation, *HR* heart rate, *RPE* rate of perceived exertion

Briefly, comparing the first and second camp, significant differences were observed in baseline sleep duration (mean difference = − 0.9 ± 0.8 h, *p* < 0.001), baseline wake time (mean difference = − 00:37 ± 01:00, *p* = 0.008), and pre-camp 800 m performance (mean difference = − 11.8 ± 17.1 s, *p* = 0.005). No significant differences were observed for baseline bedtime (mean difference = 00:09 ± 00:54, *p* = 0.422), baseline chronic training load (mean difference = 81 ± 353, *p* = 0.244), training-camp session load (mean difference = -70 ± 299, *p* = 0.237) and pre-camp 100 m performance (mean difference = − 1.5 ± 3.6 s, *p* = 0.084).

### Sleep

**Fig.4:**
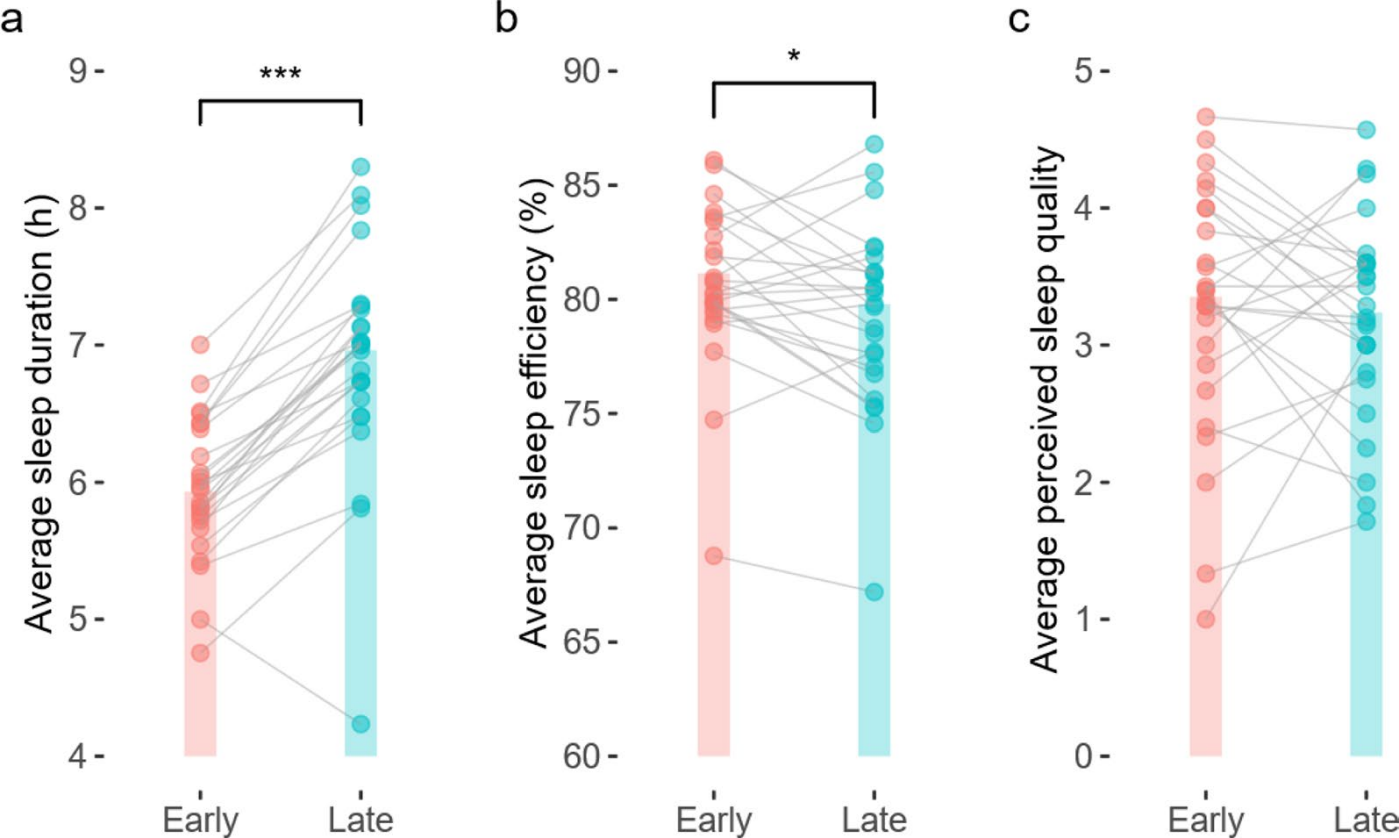
Mean values (bars) and individual data points (dots and lines) for average sleep duration (**A**), average sleep efficiency (**B**), and average perceived sleep quality (**C**) in the early (pink) and late (blue) condition. Asterisks indicate significant differences between conditions: *p < .05, ***p < .001

The effect of the condition on sleep duration is presented in Fig. 4a and is reflective of path a_1_ in Fig. 1. Sleep duration was significantly greater in the late condition compared to the early condition (early: 5.9 ± 0.6 h; late: 6.9 ± 0.8 h; mean difference: 1.0 ± 0.5 h; t = 14.0, *p* < 0.001). This increase was accompanied by a significant delay in both bedtime (*p* < 0.001), and wake-up time (*p* < 0.001), with the latter showing a larger shift (Table 1). Sleep efficiency was significantly lower in the late condition compared to the early condition (early: 81.0 ± 4.3%; late: 79.6 ± 4.4%; mean difference: –1.3 ± 3.7%; t = − 2.2, *p* = 0.03; Fig. 4b). No significant differences were observed in perceived sleep quality (early: 3.3 ± 1.0; late: 3.3 ± 0.7; mean difference = 0.0 ± 0.9; t = 0.7, *p* = 0.50; Fig. 4c) or in sleep onset latency (*p* = 0.53; Table 1).

Considering sex differences, no condition × sex interaction effects were observed (*p* > 0.12). Across both conditions, men slept significantly less than women (men: 6.1 ± 0.9 h; women: 6.7 ± 0.7 h; *p* = 0.01) and went to bed significantly later (men: 23:24 ± 0:29; women: 22:48 ± 0:26; *p* < 0.001). No differences between men and women were observed for wake-up time, sleep efficiency, perceived sleep quality, or sleep onset latency (*p* > 0.18).

The effect of condition on napping behavior is presented in Table 1. There were significantly fewer naps in the late compared to the early condition, reflecting an estimated reduction of 58% (*p* = 0.03). Nevertheless, 24-h sleep duration was significantly greater in the late condition (*p* < 0.001), with men sleeping less than women (men: 6.6 ± 0.2 h, women: 7.0 ± 0.5; *p* = 0.04). No significant difference was observed in average nap duration between conditions (*p* = 0.53) and no significant main effects of sex were observed for napping frequency and duration (*p* > 0.30).

### Recovery

Changes in heart rate and RPE during the standardized warm-up did not differ between conditions at any intensity (*p* > 0.08; Table 1). For perceived measures of recovery, there was a significant condition × day interaction on perceived emotional balance (t = 2.1, *p* = 0.04). Emotional balance declined significantly across camp days in the early condition (b = − 0.1, SE = 0.03, t = − 3.3, *p* = 0.001), but not in the late condition (b = − 0.0, SE = 0.03, t = − 0.3, *p* = 0.75). There were no significant condition x day effects for any other recovery-related items (*p* > 0.15), nor main effects of condition over the course of the training camp (*p* > 0.16; Table 1).

### Performance

As shown in Table 2, no significant effects were observed for 100 m performance (*p* > 0.29). For the 800 m, there was no significant interaction effect or main effect of condition (*p* > 0.20), but a significant main effect of timepoint indicated overall improvement from pre- to post-training camp (*p* = 0.01). Regarding motivation, no significant interaction effect or main effect of timepoint was found (*p* > 0.10); however, a significant main effect of condition indicated greater motivation in the late compared to the early condition (*p* = 0.01).

**Table 2:** Performance and motivation values across conditions (early and late) and timepoints (pre and post)

		Pre mean ± SD	Post mean ± SD	Post–Pre mean ± SD	Intervention	Timepoint	Intervention x timepoint
100 m (s)	Early	66.1 ± 5.4	65.9 ± 5.4	− 0.2 ± 1.9	F_1,19_ = 1.18 *p* = .291 pη^2^ = .06	F_1,19_ = 0.03 *p* = .858 pη^2^ < .01	F_1,19_ = 0.24 *p* = .629 pη^2^ = .01
	Late	66.9 ± 5.5	67.0 ± 6.0	0.1 ± 1.8			
800 m (s)	Early	607.6 ± 37.2	600.2 ± 36.3	− 7.4 ± 20.1	F_1,20_ = 1.72 *p* = .204 pη^2^ = .08	F_1,20_ = 7.32 *p* = .014 pη^2^ = .27	F_1,20_ = 0.02 *p* = .884 pη^2^ < .01
	Late	611.7 ± 37.7	605.2 ± 31.7	− 6.6 ± 13.5			
Motivation	Early	56.8 ± 18.6	49.3 ± 22.1	− 7.5 ± 18.7	F_1,23_ = 7.58 *p* = .011 pη^2^ = .25	F_1,23_ = 2.87 *p* = .104 pη^2^ = .11	F_1,23_ = 1.34 p = .259 pη^2^ = .06
	Late	64.1 ± 12.8	63.6 ± 20.1	− 0.5 ± 18.6			

^a^ANOVA results for the intervention × timepoint interaction and main effects of intervention and timepoint are included

^b^*SD* standard deviation, *pη*^*2*^ partial eta squared

### Mediation Analysis

**Fig.5:**
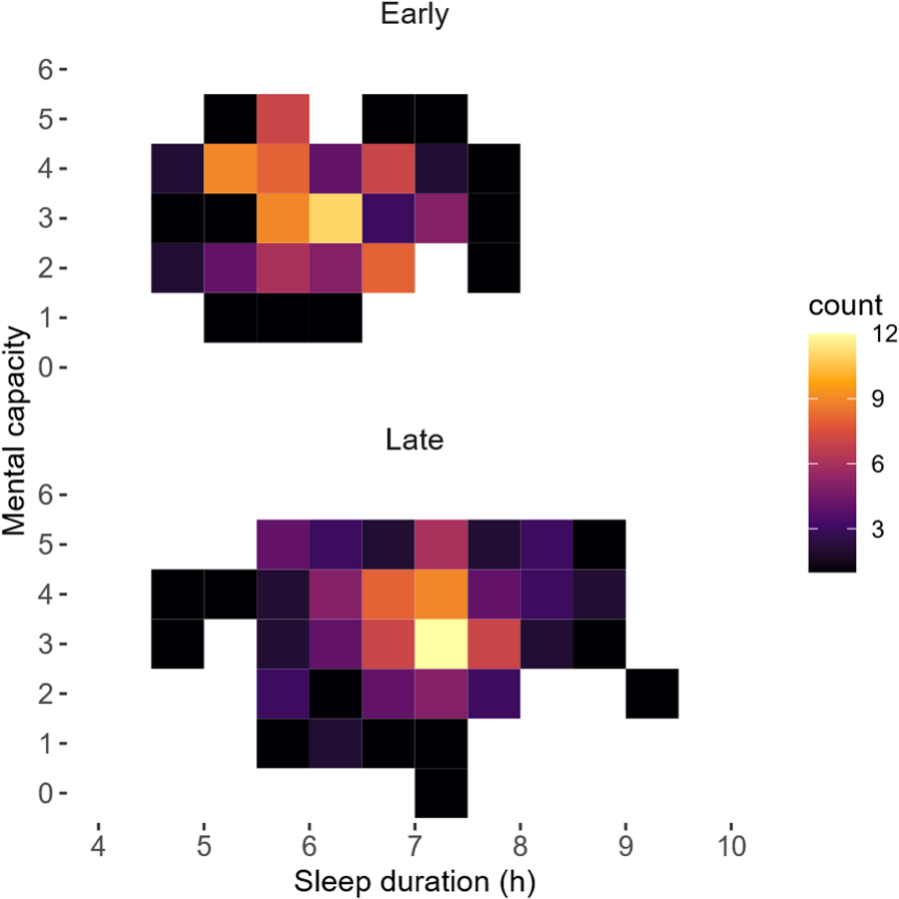
Relationship between sleep duration and mental capacity in the early (top) and late (bottom) condition. Each panel displays a 2D binned heatmap showing the distribution of observations across sleep duration and mental capacity levels. Color intensity reflects the number of observations per bin, with brighter colors indicating higher densities

The effect of the condition on mental capacity, accounting for sleep duration, is illustrated in Fig. 5. No significant association was found between sleep duration and mental capacity (b = − 0.0 SE = 0.1, t = − 0.28, *p* = 0.79; path m_1_), nor was there a significant direct effect of the condition on mental capacity after adjusting for sleep duration (b = 0.7, SE = 0.4, t = 1.81, *p* = 0.07; path a_2_), although the result approached significance. No significant condition × day effect (*p* = 0.21) was observed.

**Fig.6:**
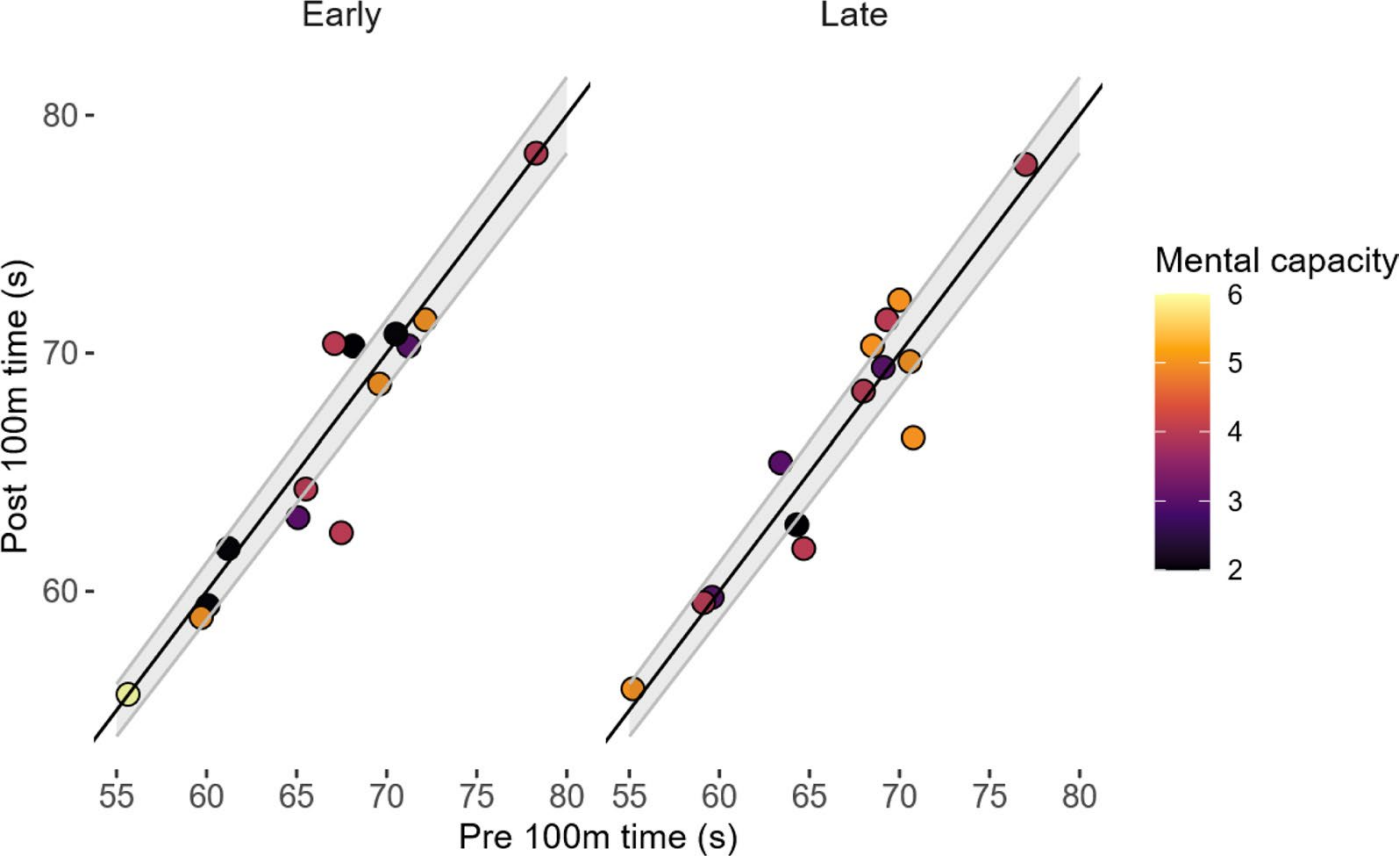
Relationship between pre- and post-100 m performance in the early (left) and late (right) condition. Each point represents an individual observation. The diagonal line indicates equal performance pre- and post-camp, with the shaded area representing a ± 2% range around this line. Points below the line indicate improved performance; points above indicate decreased performance. Color intensity corresponds to mental capacity levels, with brighter colors representing higher mental capacity

The relationship between 100 m performance after the camp, perceived mental capacity on that day, and the condition is depicted in Fig. 6. There was no significant relationship between mental capacity and 100 m performance (b = − 0.2, SE = 0.3, t = − 0.5, *p* = 0.60; path m_2_), nor was there a significant direct effect of condition on 100 m performance (b = 0.6, SE = 0.8, t = 0.8, *p* = 0.46; path c).

## Discussion

The main findings of the current study were: (1) sleep duration increased by one hour on average when training commenced at 09:00 AM compared to 07:00 AM; (2) and the decline of emotional balance was mitigated when training time was delayed; (3) however, there were no further significant effects on recovery or swimming performance; (4) sleep duration did not explain the relationship between delayed morning training and perceived mental capacity; (5) nor did perceived mental capacity explain the relationship between delayed morning training and 100 m performance.

### Sleep

Previous studies have shown that later training sessions are associated with longer sleep duration compared to earlier ones [17, 18, 20]; however, these studies used observational designs with naturally varying training times. The present study, employing an experimental design, supports these findings and extends them by providing causal evidence that delaying early morning training leads to increased sleep duration. This was the case for both men and women, although men slept significantly less than women regardless of condition, alongside later bedtimes. These findings are in line with previous research showing that males develop later bedtimes than females, with the greatest sex differences during adolescence [35, 36]. Overall, the one-hour increase observed here was insufficient for most swimmers to achieve the recommended >8 h of sleep [15, 37]. Considering that wake times in the late condition were earlier than free-day wake times reported in comparable adolescent swimmer samples [17, 38], a further delay in training start time might have resulted in additional sleep extension. Sleep efficiency was also below the recommended 85% [39], in both conditions, and decreased slightly when training was delayed, which may be related to longer sleep duration. Despite this, perceived sleep quality remained unchanged, consistent with the findings of Ashby et al. [17].

A common strategy among athletes to compensate for nocturnal sleep loss is daytime napping, which has a known potential to enhance physical performance and reduce perceived fatigue [40–42], with some evidence suggesting these benefits are more pronounced following sleep restriction [41]. The current findings suggest that swimmers indeed attempted to compensate for the reduced nocturnal sleep, reflected by 58% more naps in the early than in the late condition; however, this only slightly attenuated the difference in sleep duration between conditions.

Together these findings suggest that while delaying training start to 09:00 extends sleep duration, it may be insufficient to achieve the recommended sleep quantity and quality for adolescent swimmers—at least within the limited time frame of a training camp.

### Recovery

Although sleep duration increased by one hour when training was delayed, the impact of morning training delay on recovery was limited. While the decline in emotional balance across the training camp was mitigated, there was no further effect of delaying early morning training on other recovery measures. This was accompanied by the absence of a significant relationship between sleep duration and perceived mental capacity. These findings are in line with previous research, albeit in a very different population of adult ultra-runners, where Roberts et al. [43] reported no significant relationship between sleep duration and mental capacity the following day. On the other hand, Roberts et al.[43] did find a small yet significant relationship between sleep duration and overall recovery. To avoid fishing and inflating the type I error, the present study focused on mental capacity a priori, given the influence of sleep on attention and mood [2]. However, including an additional SRSS items in the analysis, such as emotional balance, may have yielded different results. These results suggest that sleep duration might not be as important for perceived recovery as presumed. However, it should be considered that the present study covered only a narrow range of sleep durations over a limited timespan. It therefore remains unclear whether a relationship between sleep duration and recovery would emerge across a broader range of sleep durations, at different parts of that range, or following longer-term adaptation. Future research is needed to examine the relationship between sleep and recovery across a broader spectrum of sleep durations and over longer period of time.

### Performance

The current study is the first to assess the effect of early morning training delay on swimming performance. The findings suggest that the change in performance across an 8-day training camp is not altered by delaying early morning training from 07:00 AM to 09:00 AM. In contrast, a meta-analysis by Cunha et al.[21], indicated that sleep extension of 46–113 min can improve sports performance. Additionally, improvements in motor and cognitive performance have been reported after 4–7 days of sleep extension in non-athlete populations [45–47]. This discrepancy may relate to differences in performance type, as Fullagar et al. [48] suggested that cognitively demanding tasks may be more susceptible to performance decrements upon sleep loss. Additionally, in the studies included by Cunha et al.[21], sleep extension typically resulted in total sleep durations exceeding 8 h, whereas in the present study mean sleep duration reached only 6.9 h. Moreover, unlike the studies summarized by Cunha et al., the present investigation was conducted during a period of high training load, which may have masked any potential performance benefits of sleep extension, though this remains speculative. Notably, despite the intensive camp, 100 m performance was maintained and 800 m performance even significantly improved. However, the observed improvement was within the standard error of measurement of 8.7 s previously reported for hand-timed 800 m swimming performance and should be interpreted accordingly [49]. Nonetheless, daily training load during the camp doubled compared to baseline, indicating a substantial increase in training load without any observed performance decrement. These findings suggest that swimming performance may be relatively robust to both high training load and extended sleep duration, in the short term.

In contrast to performance, motivation prior to performance testing was generally greater in the late than in the early condition. This is in line with previous research showing greater motivation following ~1.4 h sleep extension over four days [47]. Although there is also evidence for a time-of-day effect on motivation [50], the variation in test time in the current study (~ 2 h) was relatively small. Greater motivation in the late condition may have implications for training adherence and quality, and might thereby influence performance over the longer term.

### Limitations

The present study has some limitations that should be considered when interpreting the results. First, the two camps were separated by 9 months, allowing for period effects (systematic differences in the participants’ baseline state due to factors such as performance development or maturation). In fact, these period effects were observed for baseline sleep measures and 800 m performance. Although baseline values did not differ between conditions, these effects should be considered when interpreting the results. Unfortunately, a shorter time interval was not feasible, as both camps had to fall within high-training-load periods, occur in seasonally comparable periods, and align with the schedules of two national-level youth teams from different nations. Second, missing values reduced the sample size. While the study was adequately powered to detect changes in our primary outcome, sleep duration, this cannot be assumed for the secondary outcomes, for which sample sizes but also other determinants of statistical power could vary. Therefore, the respective p-values should be interpreted with explicit caution. Relatedly, napping was not included in the mediation analysis because it would have reduced the sample size and added complexity to the model. Considering that napping is known to enhance physical performance and reduce perceived fatigue [40–42], this may have influenced the results. Third, this study did not record the time of day at which the SRSS questionnaire was completed. Since time of day can influence perceived mental and physical fatigue independently of preceding sleep duration [44], this may have affected the SRSS results. However, participants were provided with a link to the questionnaire in the morning and instructed to complete it at that time. Nevertheless, future research should consider the timing of questionnaire completion. Relatedly, test times differed between conditions, which could be considered as a potential confounder of the camp-induced performance change. However, given the small discrepancy in test times, the inconsistent evidence for diurnal variation in endurance performance in the literature [51], and, in line with this, the absence of a main effect of condition on absolute performance, this is considered unlikely. Moreover, a review by Chtourou & Souissi [52], suggested that training adaptations are most apparent at the time of day at which training took place, supporting the present approach of aligning test times with training times. Additionally, the use of 10-s carotid palpation for heart rate and handheld stopwatches for swimming performance are less precise than their electronic alternatives, which should be considered when interpreting these outcomes. However, electronic timing was not available, measuring heart rate through chest-straps is impractical in men due to slippage during swimming, and neither outcome was a primary measure of the study. More broadly, the applied setting meant that conditions could not be fully standardized, potentially increasing variability and influencing the results. Nevertheless, this real-world context is also a strength of the study, enhancing its ecological validity and practical relevance. Finally, most swimmers in the current study exhibited a neutral chronotype; this limited variation precluded a meaningful analysis with chronotype as a moderator and limits the generalizability of the findings to early-birds and late-owls. It would be interesting to explore whether responses to delayed early morning training differ between chronotypes, specifically whether late-owls benefit more from delayed morning training time.

## Conclusion

The present study assessed the effect of delaying early morning training on sleep, perceived recovery, and swimming performance in high-performing adolescent swimmers. The results demonstrate that delaying morning training start from 07:00 AM to 09:00 AM increases sleep duration by one hour, but this was insufficient for swimmers to reach the recommended 8–10 h of sleep. Additionally, delaying early morning training did not significantly affect perceived recovery or swimming performance in response to the training load imposed during the camp. These findings may be explained by chronic sleep deprivation, the high training load, naps during the training camp. Moreover, the relatively short study period may have limited the opportunity for changes in performance or recovery to manifest. Nevertheless, the study provides evidence that early morning training reduces sleep duration and highlights the persistent sleep challenges faced by national-level adolescent swimmers. Coaches should plan early morning sessions carefully and, when possible, delay morning training start times to support healthy sleep in their athletes. Future research is warranted to explore strategies that enable swimmers to meet sleep recommendations and to examine the effects on recovery and performance over a longer period.

## Data Availability

The datasets generated and/or analyzed during the current study are available in the SleepTrain_analysis repository on GitHub, https://github.com/MBrandts98/SleepTrain_analysis.

## Abbreviations

ANOVA: Analysis of variance
DAG: Directed acyclic graph
RPE: Rate of perceived exertion
SE: Standard error
sRPE: Session rate of perceived exertion
SRSS: Short Recovery and Stress Scale

## Code Availability

All analysis code is openly available on GitHub: https://github.com/MBrandts98/SleepTrain_analysis.

## References

[1] Zielinski MR, McKenna JT, McCarley RW. Functions and mechanisms of sleep. AIMS Neurosci. 2016;3:67–104. 10.3934/Neuroscience.2016.1.67. 28413828 10.3934/Neuroscience.2016.1.67 PMC5390528

[2] Krause AJ, Simon EB, Mander BA, Greer SM, Saletin JM, Goldstein-Piekarski AN, et al. The sleep-deprived human brain. Nat Rev Neurosci. 2017;18:404–18. 10.1038/nrn.2017.55. 28515433 10.1038/nrn.2017.55 PMC6143346

[3] Garbarino S, Lanteri P, Bragazzi NL, Magnavita N, Scoditti E. Role of sleep deprivation in immune-related disease risk and outcomes. Commun Biol. 2021;4:1304. 10.1038/s42003-021-02825-4. 34795404 10.1038/s42003-021-02825-4 PMC8602722

[4] Lamon S, Morabito A, Arentson‐Lantz E, Knowles O, Vincent GE, Condo D, et al 2021 The effect of acute sleep deprivation on skeletal muscle protein synthesis and the hormonal environment. Physiol Rep 10.14814/phy2.14660 10.14814/phy2.14660 PMC7785053 33400856

[5] Reynolds AC, Banks S. Total sleep deprivation, chronic sleep restriction and sleep disruption. Prog Brain Res. 2010;185:91–103. 10.1016/B978-0-444-53702-7.00006-3. 21075235 10.1016/B978-0-444-53702-7.00006-3

[6] Craven J, Roberts L, McCartney D, Desbrow B, Sabapathy S, Bellinger P, et al. Effects of acute sleep loss on physical performance: a systematic and meta-analytical review. Sports Med. 2022. 10.1007/s40279-022-01706-y. 35708888 10.1007/s40279-022-01706-y PMC9584849

[7] Gong M, Sun M, Sun Y, Jin L, Li S. Effects of Acute Sleep Deprivation on Sporting Performance in Athletes: A Comprehensive Systematic Review and Meta-Analysis. Nat Sci Sleep. 2024;16:935–48. 10.2147/NSS.S467531. 39006249 10.2147/NSS.S467531 PMC11246080

[8] Kong Y, Yu B, Guan G, Wang Y, He H. Effects of sleep deprivation on sports performance and perceived exertion in athletes and non-athletes: a systematic review and meta-analysis. Front Physiol. 2025;16:1544286. 10.3389/fphys.2025.1544286. 40236824 10.3389/fphys.2025.1544286 PMC11996801

[9] Rae DE, Chin T, Dikgomo K, Hill L, McKune AJ, Kohn TA, et al. One night of partial sleep deprivation impairs recovery from a single exercise training session. Eur J Appl Physiol. 2017;117:699–712. 10.1007/s00421-017-3565-5. 28247026 10.1007/s00421-017-3565-5

[10] Braun-Trocchio R, Graybeal AJ, Kreutzer A, Warfield E, Renteria J, Harrison K, et al. Recovery strategies in endurance athletes. J Funct Morphol Kinesiol. 2022;7:22. 10.3390/jfmk7010022. 35225908 10.3390/jfmk7010022 PMC8883945

[11] Venter R. Perceptions of team athletes on the importance of recovery modalities. Eur J Sport Sci. 2014;14:S69-76. 10.1080/17461391.2011.643924. 24444246 10.1080/17461391.2011.643924

[12] Shell SJ, Slattery K, Clark B, Broatch JR, Halson S, Kellmann M, et al. Perceptions and use of recovery strategies: do swimmers and coaches believe they are effective? J Sports Sci. 2020;38:2092–9. 10.1080/02640414.2020.1770925. 32475220 10.1080/02640414.2020.1770925

[13] Vlahoyiannis A, Aphamis G, Bogdanis GC, Sakkas GK, Andreou E, Giannaki CD. Deconstructing athletes’ sleep: a systematic review of the influence of age, sex, athletic expertise, sport type, and season on sleep characteristics. J Sport Health Sci. 2020;10:387–402. 10.1016/j.jshs.2020.03.006. 32325024 10.1016/j.jshs.2020.03.006 PMC8343120

[14] Leeder J, Glaister M, Pizzoferro K, Dawson J, Pedlar C. Sleep duration and quality in elite athletes measured using wristwatch actigraphy. J Sports Sci. 2012;30:541–5. 10.1080/02640414.2012.660188. 22329779 10.1080/02640414.2012.660188

[15] Hirshkowitz M, Whiton K, Albert SM, Alessi C, Bruni O, DonCarlos L, et al. National Sleep Foundation’s updated sleep duration recommendations: final report. Sleep Health. 2015;1:233–43. 10.1016/j.sleh.2015.10.004. 29073398 10.1016/j.sleh.2015.10.004

[16] Sargent C, Lastella M, Halson SL, Roach GD. How Much Sleep Does an Elite Athlete Need. Int J Sports Physiol Perform. 2021;16:1–12. 10.1123/ijspp.2020-0896. 34021090 10.1123/ijspp.2020-0896

[17] Ashby C, Driller MW, Suppiah HT, O’Donnell S 2024 Sink or Swim? Sleep Patterns in Highly Trained Adolescent Swimmers during the In-Season Phase of Training. Sleep Sci. 10.1055/s-0043-1777778 10.1055/s-0043-1777778 PMC11152630 38846587

[18] Gudmundsdottir SL. Training schedule and sleep in adolescent swimmers. Pediatr Exerc Sci. 2020;32:16–22. 10.1123/pes.2019-0067. 31592774 10.1123/pes.2019-0067

[19] Walsh JA, Sanders D, Hamilton DL, Walshe I. Sleep profiles of elite swimmers during different training phases. J Strength Cond Res. 2019;33:811–8. 10.1519/jsc.0000000000002866. 30289871 10.1519/JSC.0000000000002866

[20] Sargent C, Halson S. Roach GD 2024 Sleep or swim? Early-morning training severely restricts the amount of sleep obtained by elite swimmers. Eur J Sport Sci. 2012. 10.1080/17461391696711. 24444223 10.1080/17461391.2012.696711

[21] Cunha LA, Costa J, Marques EA, Brito J, Lastella M, Figueiredo P 2023 The Impact of Sleep Interventions on Athletic Performance: A Systematic Review. Sports Med – Open 10.1186/s40798-023-00599-z 10.1186/s40798-023-00599-z PMC10354314 37462808

[22] Hausswirth C, Louis J, Aubry A, Bonnet G, Duffield R, Le Meur Y. Evidence of Disturbed Sleep and Increased Illness in Overreached Endurance Athletes. Med Sci Sports Exerc. 2014;46:1036–45. 10.1249/mss.0000000000000177. 24091995 10.1249/MSS.0000000000000177

[23] Schaal K, Le Meur Y, Louis J, Filliard J-R, Hellard P, Casazza GA, et al. Whole-Body Cryostimulation Limits Overreaching in Elite Synchronized Swimmers. Med Sci Sports Exerc. 2015;47:1416–25. 10.1249/mss.0000000000000546. 25314578 10.1249/MSS.0000000000000546

[24] Roberts SSH, Wei-Peng T, Warmington SA. Effects of training and competition on the sleep of elite athletes: a systematic review and meta-analysis. Br J Sports Med. 2019;53:513–22. 10.1136/bjsports-2018-099322. 30217831 10.1136/bjsports-2018-099322

[25] Fullagar HHK, Sampson JA, Delaney JA, McKay BA, Murray A. The relationship between objective measures of sleep and training load across different phases of the season in American collegiate football players. Sci Med Footb. 2019;3:326–32. 10.1080/24733938.2019.1618491.

[26] McKay AKA, Stellingwerff T, Smith ES, Martin DT, Mujika I, Goosey-Tolfrey VL, et al. Defining training and performance caliber: a participant classification framework. Int J Sports Physiol Perform. 2022;17:317–31. 10.1123/ijspp.2021-0451. 34965513 10.1123/ijspp.2021-0451

[27] Horne JA, Ostberg O. A self-assessment questionnaire to determine morningness–eveningness in human circadian rhythms. Int J Chronobiol. 1976;4:97–110. 1027738

[28] Lamberts RP, Swart J, Noakes TD, Lambert MI. A novel submaximal cycle test to monitor fatigue and predict cycling performance. Br J Sports Med. 2011;45:797–804. 10.1136/bjsm.2009.061325. 19622525 10.1136/bjsm.2009.061325

[29] Migueles JH, Rowlands AV, Huber F, Sabia S, Van Hees VT. GGIR: a research community–driven open source R package for generating physical activity and sleep outcomes from multi-day raw accelerometer data. J Meas Phys Behav. 2019;2:188–96. 10.1123/jmpb.2018-0063.

[30] Plekhanova T, Rowlands AV, Davies MJ, Hall AP, Yates T, Edwardson CL. Validation of an automated sleep detection algorithm using data from multiple accelerometer brands. J Sleep Res. 2023;32:e13760. 10.1111/jsr.13760. 36317222 10.1111/jsr.13760

[31] Van Hees VT, Sabia S, Anderson KN, Denton SJ, Oliver J, Catt M, et al. A novel, open access method to assess sleep duration using a wrist-worn accelerometer. PLoS ONE. 2015;10:e0142533. 10.1371/journal.pone.0142533. 26569414 10.1371/journal.pone.0142533 PMC4646630

[32] Van Hees VT, Sabia S, Jones SE, Wood AR, Anderson KN, Kivimäki M, et al. Estimating sleep parameters using an accelerometer without sleep diary. Sci Rep. 2018;8:12975. 10.1038/s41598-018-31266-z. 30154500 10.1038/s41598-018-31266-z PMC6113241

[33] Hitzschke B, Kölling S, Ferrauti A, Meyer T, Pfeiffer M, Kellmann M. Entwicklung der Kurzskala zur Erfassung von Erholung und Beanspruchung im Sport (KEB). Z Sportpsychol. 2015;22:146–62. 10.1026/1612-5010/a000150.

[34] Hecksteden A, Schmartz GP, Egyptien Y, Aus Der Fünten K, Keller A, Meyer T. Forecasting football injuries by combining screening, monitoring and machine learning. Sci Med Footb. 2023;7:214–28. 10.1080/24733938.2022.2095006. 35757889 10.1080/24733938.2022.2095006

[35] Roenneberg T, Kuehnle T, Pramstaller PP, Ricken J, Havel M, Guth A, et al. A marker for the end of adolescence. Curr Biol. 2004;14:R1038–9. 10.1016/j.cub.2004.11.039. 15620633 10.1016/j.cub.2004.11.039

[36] Fischer D, Lombardi DA, Marucci-Wellman H, Roenneberg T 2017 Chronotypes in the US – Influence of age and sex. Tosini G, editor. PLOS ONE. 12 e0178782 10.1371/journal.pone.0178782 10.1371/journal.pone.0178782 PMC5479630 28636610

[37] Roberts SSH, Teo W-P, Aisbett B, Warmington SA. Extended sleep maintains endurance performance better than normal or restricted sleep. Med Sci Sports Exerc. 2019;51:2516–23. 10.1249/MSS.0000000000002071. 31246714 10.1249/MSS.0000000000002071

[38] Steenekamp T, Zaslona J, Gander P, Rowlands D, Leigh Signal T. Sleep/wake behaviour of competitive adolescent athletes in New Zealand: insight into the impact of early morning training. Sleep Med. 2021;77:88–95. 10.1016/j.sleep.2020.11.023. 33341643 10.1016/j.sleep.2020.11.023

[39] Ohayon M, Wickwire EM, Hirshkowitz M, Albert SM, Avidan A, Daly FJ, et al. National Sleep Foundation’s sleep quality recommendations: first report. Sleep Health. 2017;3:6–19. 10.1016/j.sleh.2016.11.006. 28346153 10.1016/j.sleh.2016.11.006

[40] Teece A, Beaven CM, Suppiah H, Argus CK, Gill N, Driller MW. Routine, routine, routine: sleep regularity and its association with sleep metrics in professional rugby union athletes. Sports Med - Open. 2024;10:51. 10.1186/s40798-024-00709-5. 38722443 10.1186/s40798-024-00709-5 PMC11082106

[41] Lastella M, Halson SL, Vitale JA, Memon AR, Vincent GE. To Nap or Not to Nap? A Systematic Review Evaluating Napping Behavior in Athletes and the Impact on Various Measures of Athletic Performance. Nat Sci Sleep. 2021;13:841–62. 10.2147/nss.s315556. 34194254 10.2147/NSS.S315556 PMC8238550

[42] Mesas AE, Núñez De Arenas-Arroyo S, Martinez-Vizcaino V, Garrido-Miguel M, Fernández-Rodríguez R, Bizzozero-Peroni B, et al. Is daytime napping an effective strategy to improve sport-related cognitive and physical performance and reduce fatigue? A systematic review and meta-analysis of randomised controlled trials. Br J Sports Med. 2023;57:417–26. 10.1136/bjsports-2022-106355. 36690376 10.1136/bjsports-2022-106355

[43] Roberts SSH, Main LC, Condo D, Carr A, Jardine W, Urwin C, et al. Sex differences among endurance athletes in the pre-race relationships between sleep, and perceived stress and recovery. J Sports Sci. 2022;40:1542–51. 10.1080/02640414.2022.2091345. 35767576 10.1080/02640414.2022.2091345

[44] Kline CE, Durstine JL, Davis JM, Moore TA, Devlin TM, Zielinski MR, et al. Circadian variation in swim performance. J Appl Physiol. 2007;102:641–9. 10.1152/japplphysiol.00910.2006. 17095634 10.1152/japplphysiol.00910.2006

[45] Kamdar BB, Kaplan KA, Kezirian EJ, Dement WC. The impact of extended sleep on daytime alertness, vigilance, and mood. Sleep Med. 2004;5:441–8. 10.1016/j.sleep.2004.05.003. 15341888 10.1016/j.sleep.2004.05.003

[46] Parsons CE, Young KS. Beneficial effects of sleep extension on daily emotion in short-sleeping young adults: An experience sampling study. Sleep Health. 2022;8:505–13. 10.1016/j.sleh.2022.05.008. 35872150 10.1016/j.sleh.2022.05.008

[47] Ritland BM, Simonelli G, Gentili RJ, Smith JC, He X, Mantua J, et al. Effects of sleep extension on cognitive/motor performance and motivation in military tactical athletes. Sleep Med. 2019;58:48–55. 10.1016/j.sleep.2019.03.013. 31096123 10.1016/j.sleep.2019.03.013

[48] Fullagar HHK, Vincent GE, McCullough M, Halson S, Fowler P. Sleep and sport performance. J Clin Neurophysiol. 2023;40:408–16. 10.1097/WNP.0000000000000638. 36930212 10.1097/WNP.0000000000000638

[49] Skorski S, Faude O, Rausch K, Meyer T. Reproducibility of pacing profiles in competitive swimmers. Int J Sports Med. 2012;34:152–7. 10.1055/s-0032-1316357. 22972249 10.1055/s-0032-1316357

[50] Blazer HJ, Jordan CL, Pederson JA, Rogers RR, Williams TD, Marshall MR, et al. Effects of time-of-day training preference on resistance-exercise performance. Res Q Exerc Sport. 2021;92:492–9. 10.1080/02701367.2020.1751032. 32633217 10.1080/02701367.2020.1751032

[51] Knaier R, Qian J, Roth R, Infanger D, Notter T, Wang W, et al. Diurnal variation in maximum endurance and maximum strength performance: a systematic review and meta-analysis. Med Sci Sports Exerc. 2022;54:169–80. 10.1249/MSS.0000000000002773. 34431827 10.1249/MSS.0000000000002773 PMC10308487

[52] Chtourou H, Souissi N. The effect of training at a specific time of day: a review. J Strength Cond Res. 2012;26:1984–2005. 10.1519/JSC.0b013e31825770a7. 22531613 10.1519/JSC.0b013e31825770a7

